# Exploring the role of vascular frailty in understanding blood pressure variability and mortality risk among end‐stage kidney disease patients undergoing hemodialysis

**DOI:** 10.1002/clc.24281

**Published:** 2024-05-13

**Authors:** Szu‐Ying Lee, Chia‐Ter Chao

**Affiliations:** ^1^ Division of Nephrology, Department of Internal Medicine National Taiwan University Hospital Yunlin Branch Yunlin Taiwan; ^2^ Department of Internal Medicine Min‐Sheng General Hospital Taoyuan City Taiwan; ^3^ Division of Nephrology, Department of Internal Medicine National Taiwan University Hospital and National Taiwan University College of Medicine Taipei Taiwan; ^4^ Graduate Institute of Toxicology National Taiwan University College of Medicine Taipei Taiwan

To the Editor,

Dong et al. investigated the relationship between interdialytic home blood pressure variability (BPV) and mortality in individuals with end‐stage kidney disease (ESKD) undergoing hemodialysis.^1^ Their study revealed a notable correlation between increasing home BPV and a stepwise escalation in the risk of all‐cause mortality among this population. In the discussion, the authors attributed this association to comorbid cardiovascular diseases, diabetes, systemic inflammation, and higher vascular stiffness. We believe that a more comprehensive characterization of vascular integrity impairment is needed to explain this BPV‐outcome association.

Our team previously introduced the concept of vascular frailty, which elucidates vascular structural and functional degeneration leading to physical frailty or the coexistence of both phenotypes.^2^ Vascular tissues are exquisitely susceptible to adverse consequences related to tissue senescence from chronological aging and metabotoxic stimuli, particularly uremic toxins. Vascular frailty manifests through structural degenerations such as vascular calcification and atherosclerosis, as well as functional perturbations like increased stiffness and vasomotor dysfunction. Uremic toxins predispose individuals with chronic kidney disease to developing physical frailty^3^ and contribute to the pathogenesis of vascular frailty by inducing vascular calcification and increasing vascular stiffness,^4^ thereby leading to higher BPV. It is widely acknowledged that ESKD patients are prone to developing vascular calcification over time. Moreover, individuals with ESKD exhibit accelerated aging and heightened tissue wear, culminating in physical frailty that significantly elevates mortality risk. Our previous findings demonstrated that ESKD patients with frailty faced a significantly higher risk of mortality compared with those without.^5^

From this perspective, we recommend that the authors consider incorporating additional features of vascular frailty, such as measurements of vascular calcification severity and evidence of autonomic dysfunction, in their assessments of these patients. Doing so will facilitate a more thorough understanding of the mechanisms underlying the BPV‐outcome association.

## AUTHOR CONTRIBUTIONS

Szu‐Ying Lee and Chia‐Ter Chao are responsible for drafting of this manuscript.

## CONFLICT OF INTEREST STATEMENT

The authors declare no conflict of interest.

## Data Availability

Data sharing is not applicable to this article as no new data were created or analyzed in this study.
